# Septins of Platyhelminths: Identification, Phylogeny, Expression and Localization among Developmental Stages of *Schistosoma mansoni*

**DOI:** 10.1371/journal.pntd.0002602

**Published:** 2013-12-19

**Authors:** Ana E. Zeraik, Gabriel Rinaldi, Victoria H. Mann, Anastas Popratiloff, Ana P. U. Araujo, Ricardo DeMarco, Paul J. Brindley

**Affiliations:** 1 Departamento de Física e Informática, Instituto de Física de São Carlos, Universidade de São Paulo, São Carlos, São Paulo, Brazil; 2 Department of Microbiology, Immunology & Tropical Medicine, and Research Center for Neglected Tropical and Infectious Diseases of Poverty, School of Medicine & Health Sciences, The George Washington University, Washington, D.C., United States of America; 3 Departamento de Genética, Facultad de Medicina, Universidad de la República (UDELAR), Montevideo, Uruguay; 4 Center for Microscopy and Image Analysis, The George Washington University, Washington, D.C., United States of America; Rush University Medical Center, United States of America

## Abstract

Septins are a family of eukaryotic GTP binding proteins conserved from yeasts to humans. Originally identified in mutants of budding yeast, septins participate in diverse cellular functions including cytokinesis, organization of actin networks, cell polarity, vesicle trafficking and many others. Septins assemble into heteroligomers to form filaments and rings. Here, four septins of *Schistosoma mansoni* are described, which appear to be conserved within the phylum Platyhelminthes. These orthologues were related to the SEPT5, SEPT10 and SEPT7 septins of humans, and hence we have termed the schistosome septins *Sm*SEPT5, *Sm*SEPT10, *Sm*SEPT7.1 and *Sm*SEPT7.2. Septin transcripts were detected throughout the developmental cycle of the schistosome and a similar expression profile was observed for septins in the stages examined, consistent with concerted production of these proteins to form heterocomplexes. Immunolocalization analyses undertaken with antibodies specific for *Sm*SEPT5 and *Sm*SEPT10 revealed a broad tissue distribution of septins in the schistosomulum and colocalization of septin and actin in the longitudinal and circular muscles of the sporocyst. Ciliated epidermal plates of the miracidium were rich in septins. Expression levels for these septins were elevated in germ cells in the miracidium and sporocyst. Intriguingly, septins colocalize with the protonephridial system of the cercaria, which extends laterally along the length of this larval stage. Together, the findings revealed that schistosomes expressed several septins which likely form filaments within the cells, as in other eukaryotes. Identification and localization demonstrating a broad distribution of septins across organs and tissues of schistosome contributes towards the understanding of septins in schistosomes and other flatworms.

## Author Summary

*Schistosoma mansoni* is one of the causative agents of schistosomiasis, a neglected tropical disease affecting over 230 million people in the developing world. Research on new therapies for this parasitic disease has been facilitated by the recent publication of a curated draft sequence of the schistosome genome. Here, we describe proteins from the septin family found in the genome of *S. mansoni*. The septins are increasingly recognized as central components of the cytoskeleton of eukaryotic cells. They are linked to numerous cellular functions, although the precise role(s) of these proteins is not fully understood. Schistosome septins were seen in the miracidium and sporocyst larval stages, on superficial structures, within epidermal plates and in muscles. Notably, septins were prominently expressed in the germ cells of larval stages of the blood fluke. In addition, septins were ubiquitously immuno-localized throughout the organs and tissues of the schistosomulum stage of the parasite. This is the first report on septins in schistosomes; these proteins are broadly distributed among organs and tissues of the parasite where they likely perform diverse functions. Identification and localization demonstrating a broad distribution of septins across organs and tissues of schistosome contributes towards the understanding of septins in schistosomes and other flatworms.

## Introduction

Septins comprise a family of evolutionarily highly conserved cytoskeletal proteins [1]. Absent from higher plants but otherwise ubiquitous in eukaryotes [2], [3], septins have been well characterized in human cells and model invertebrates including *Caenorhabditis elegans* and *Drosophila melanogaster* [4]–[6]. The septin family belongs to the guanosine triphosphate (GTP)ase superclass of P-loop nucleoside triphosphate (NTP)ases [1]. It was first identified due to defective cell-cycle progression in yeasts [7]. The functions attributed to septins are expanding, but span cytokinesis [7], vesicle trafficking [8], vesicle fusion [9], axonal guidance and migration [10], diffusion barriers, scaffolds [11]–[13], pathogenesis [14], [15] and others [16]. Septin function generally depends on self-assembly into hetero-oligomeric complexes, which assemble subsequently into higher-order structures such as filaments and rings [17], [18]. The septins have been consolidated as new cytoskeleton components [17].

The diversity and the number of septin-encoding genes diverge among species, ranging from one in algae [2] to 13 in humans [19]. Based on phylogenetic analysis, the metazoan septins can be classified into four groups, termed SEPT6, SEPT7, SEPT2 and SEPT3 [20]. Septins form filaments, which are composed of hetero-oligomeric complexes of septins from different groups. It has been postulated that each position of the hetero-oligomeric complex is specifically occupied by a septin group member and those cannot be replaced by member of another group [4]. Diversity among human members within most of the groups allows a multiplicity of potential complexes of human septins, with the permutation of different members of a same group in each of the hetero-oligomeric positions of the complex. However, an exception is the SEPT7 group which displays a single representative in the human genome and therefore its single member may not be replaced [4].

Schistosomiasis is considered the most important of the human helminth diseases in terms of morbidity and mortality (see [21]). Unusual among flatworms, schistosomes are dioecious, with sexual dimorphism and division of labor between sexes in the adult developmental stage [22]. Draft genome sequences for the three major species of schistosomes parasitizing humans are available [23]–[25]. Among these genomes, we identified putative septin-encoding sequences of *Schistosoma mansoni* and reported here four schistosome septins termed *Sm*SEPT5, *Sm*SEPT10, *Sm*SEPT7.1, and *Sm*SEPT7.2, based on sequence identity with numbered human orthologues. Phylogenetic analyses in tandem with expression profiles of transcripts among developmental stages point to structural roles for these septin-like proteins in this pathogen. Confocal imaging revealed tissue-specific and/or ubiquitous localization of septins, suggesting specialized functions in schistosomes, and in flatworms at large, in addition to cytokinesis. To our knowledge, this is the first report of septins in any member of the phylum Platyhelminthes, or indeed in any Lophotrochozoan - a major evolutionary branch of the Bilateria [26].

## Materials and Methods

### Ethics statement

Mice infected with *S. mansoni* were obtained from the Biomedical Research Institute (BRI), Rockville, MD and housed at the Animal Research Facility of the George Washington University Medical School, which is accredited by the American Association for Accreditation of Laboratory Animal Care (AAALAC no. 000347) and has an Animal Welfare Assurance on file with the National Institutes of Health, Office of Laboratory Animal Welfare, OLAW assurance number A3205-01. All procedures employed were consistent with the Guide for the Care and Use of Laboratory Animals. Maintenance of the mice and recovery of schistosomes were approved by the Institutional Animal Care and Use Committee of the George Washington University. Procedures used for the production of antibodies were performed in accordance with the National Research Council's guide for care and use of laboratory animals [27].

### Developmental stages of *Schistosoma mansoni*

*Biomphalaria glabrata* snails and Swiss-Webster mice infected with the NMRI (Puerto Rican) strain of *S. mansoni* were supplied by Drs. Fred Lewis and Matt Tucker, Biomedical Research Institute, Rockville, MD under NIH-NIAID contract HHSN272201000005I. Developmental stages of schistosomes were obtained as described [28]–[30]. In brief, adult developmental stages of the worms were recovered from infected mice by portal perfusion. Eggs were isolated from livers of schistosome-infected mice and newly hatched miracidia obtained by hatching these eggs. Primary sporocysts were obtained by transferring miracidia into sporocyst medium, as described [28] and cultured for two days [28]. Cercariae released from infected snails were snap frozen at −80°C or transformed mechanically into schistosomula which were cultured in Basch's medium [31] at 37°C under 5% CO_2_ in air.

### Bioinformatics and sequence analysis

**Figure 1:**
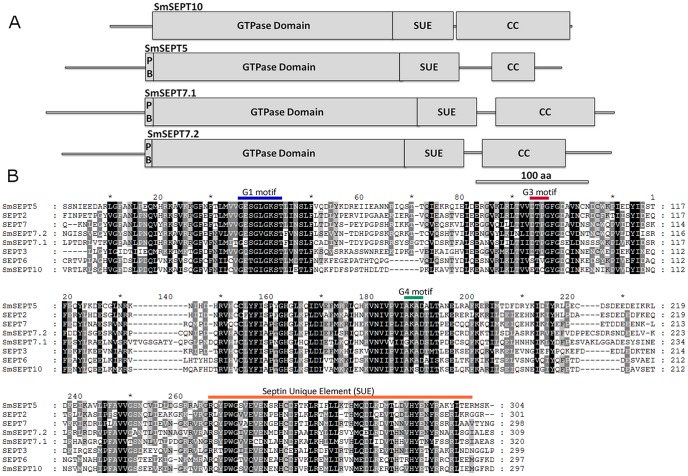
Conservation of schistosome septins. Panel A: Schematic representation of structure of *Schistosoma mansoni* septins – locations of PB, polybasic region; GTPase domain; SUE, Septin Unique Element; and CC, coiled coil structures, are indicated. B: Multiple sequence alignment of the four *S. mansoni* septins and representatives of the four groups of human septins. Amino acids that are identical or similar are shaded in black and grey, respectively. Characteristic motifs are indicated and amino acid positions numbered.

**Figure 2:**
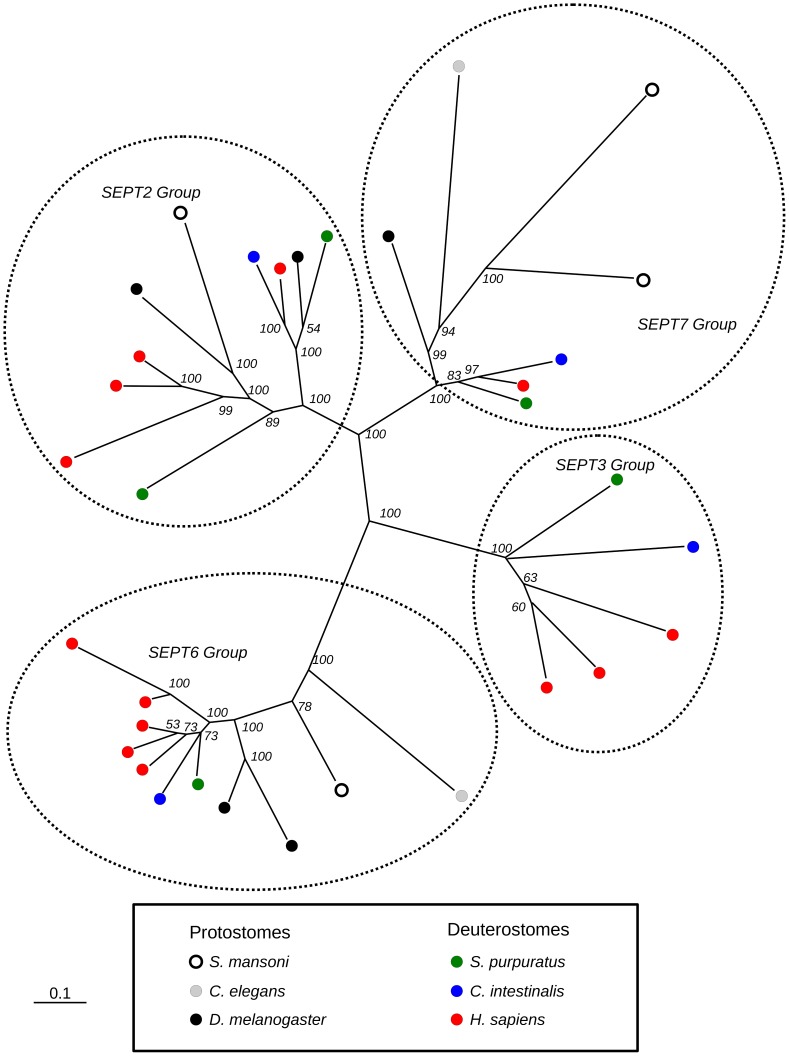
Evolutionary relationship among septins of humans and *Schistosoma mansoni*. Phylogenetic tree (based on Bayesian inference) generated from a multiple alignment of the conserved GTPase domains of septins from *S. mansoni* and two other informative protostomes and three deuterostomes. The numbers on the tree nodes are posterior probabilities calculated by MrBayes. Branches with the four discrete groups of septins are enclosed by the dotted lines. Species are identified by the small circles of different shapes and colors as indicated in the lower panel.

Coding regions deduced in the genome of *Schistosoma mansoni* [23], [32] were used as a database to identify septin genes through the tBLASTn program, using all human septins as queries. Four putative orthologues of septin were identified: Smp_041060, Smp_060070, Smp_003620 and Smp_029890. The multiple sequence alignment of the GTPase domain from these four *S. mansoni* septins with septins from *Homo sapiens*, *Caenorhabditis elegans*, *Drosophila melanogaster*, *Strongylocentrotus purpuratus* and *Ciona intestinalis* was accomplished using ClustalX2 [33]. Additional alignment was performed with GTPases domains from several platyhelminths using the same approach. Phylogenetic analyses were performed using a Bayesian inference method implemented in MrBayes (v3.1.2) [34]. All analyses were run using default parameters, except by the use of the command “prset aamodelpr = mixed”, which allows the use of a mixture of amino acid models with fixed rate to estimate the appropriate model for the analysis. Analyses were stopped after 1,000,000 generations, with samplings every one hundredth generation. Tree information was summarized utilizing the “sumt burnin = 2500”, which discards the first 250,000 generations. In all cases, the measured potential scale reduction factor (PSRF), obtained using the “sump burnin = 2500” command, was equal to 1, indicating a convergence of the analysis. Amino acids models chosen by the program for each tree were: Tree 1 (Figs. 1, 2), WAG (posterior probability = 1.0); Tree 2 (Figure S1), WAG (posterior probability = 0.877) and Jones (posterior probability = 0.123). The resulting tree together containing the posterior probability for each branch was visualized using TreeView [35].

### Recombinant expression of schistosome septins; anti-septin antisera

Full length transcripts encoding the septins *Sm*SEPT5 and *Sm*SEPT10 of *S. mansoni* were amplified by PCR using the following primers: *SmSept5* forward primer, 5′-GCTAGCATG GCA AAT ATT CCG CGT TTT GG-3′; *SmSept5* reverse primer 5′-GGATCCTCAAGACGCTTGTTGACCAGTTAC-3′; *SmSept10* forward primer 5′-GCTAGCATGACTGCAGATGTTCTAAAAGCATTG-3′; *SmSept10* reverse primer ACTAGCTGTACTCTCGTCAGGATCCTTATTTCC-3′ (restriction enzyme sites underlined). Reverse transcription from total mRNAs from mixed sex adult schistosomes (BH, a Brazilian strain) was accomplished and cDNA served as template for PCRs using the primers above. Amplicons of expected sizes were ligated into pTZ57R/T (Thermo Scientific), integrity of the inserts confirmed by nucleotide sequencing (3130 Genetic Analyzer, Applied Biosystems), and the septin-encoding sequences sub-cloned into the expression vector pET28a(+) (Novagen), which introduces a His-Tag at the N-terminus of the polypeptide. Recombinant septins were expressed in *E. coli* Rosetta (DE3) strain cells transformed with pET28 constructs, with expression induced by IPTG at 0.4 mM in LB medium for 16 h at 18°C with shaking.

Rosetta cells were lysed by sonication in 50 mM Tris-HCl pH 8.0, 800 mM NaCl, 10% glycerol, 10 mM β-mercaptoethanol (buffer A), after which lysates were clarified by centrifugation at 20,000 g for 30 min. Supernatants were fractionated by affinity chromatography on Ni-NTA resin (Qiagen) equilibrated in buffer A. Immobilized proteins were eluted in 50 mM Tris-HCl pH 8.0, 300 mM NaCl, 500 mM imidazole, 10% glycerol, 10 mM, β-mercaptoethanol (buffer B). Subsequently, eluates were separated through a column of Superdex 200 10/300 GL resin (GE Healthcare Life Sciences) fitted to a liquid chromatography system (AKTA purifier, GE Healthcare Life Sciences). Purity of eluates was assessed by Coomassie stained SDS-PAGE.

Polyclonal antibodies against recombinant *Sm*SEPT5 and *Sm*SEPT10 were raised in mice. Anti-septin immunoglobulins were isolated from mouse sera by affinity chromatography using immobilized recombinant schistosome septins conjugated to HiTrap NHS resin (GE Healthcare Life Sciences). Western blot analysis was employed to examine the specificity of antibodies to recombinant *Sm*SEPT5, *Sm*SEPT10 (each at 0.5 µM) and proteins extracted from mixed sex, adult worms (50 mg). After resolution by SDS-PAGE, proteins were transferred to nitrocellulose using the Trans-blot Semi-dry Transfer cell (Bio-Rad), 30 min at 10 V. Membranes were washed three times (15 min each) with TBS-T (10 mM Tris, 150 mM NaCl, 0.1% Tween-20) and incubated with blocking buffer (5% nonfat milk powder in TBS-T) for 2 h at 4°C. Incubation with the primary antibody, anti-*Sm*SEPT5 or anti-*Sm*SEPT10 diluted 1∶1,000 in TBS-T was performed for 2 h at 4°C, followed by washes as above. Thereafter, membranes were probed with goat anti-mouse IgG (whole molecule)-alkaline phosphatase (Sigma-Aldrich), diluted 1∶5,000 in TBS-T, for 2 h at 4°C, and washed as above. Signals were developed using Bio-Rad's Alkaline Phosphatase Conjugate Substrate Kit after which membranes were photographed.

### Expression profiles of schistosome septins

Total RNA was recovered from developmental stages of schistosomes using the RNAqueous-4PCR system (Ambion). Any residual DNA in the RNA was removed by digestion with DNase (TurboDNase, Ambion). RNA concentration, purity and integrity were determined by Nanodrop 1000 spectrophotometer and Agilent 2100 Bioanalyzer; cDNA was synthesized from 150 ng RNA using the iScript cDNA Synthesis Kit (Bio-Rad). Quantitative polymerase chain reaction (qPCR) employing iQ SYBR Green Supermix (Bio-Rad), each primer at 0.3 µM in 20 µl reaction volume, was performed in a thermocycler (iCycler, Bio-Rad) fitted with real time detector (Bio-Rad iQ5). Septins-specific primers that spanned predicted exon junctions were designed as follows: *SmSept5* forward primer, 5′-GGAACTGGCTTTGAGGCTATTG-3′; *SmSept5* reverse primer, 5′-TGTTCTTGCATTTTACTCATTAGTTGTTG-3′; *SmSept10* forward primer, 5′-CGACGTCAACGCTTAATCGA-3′; *SmSept10* reverse primer 5′-CTTTAACACGCTGAACAAACATTTG-3′; *SmSept7.1* forward primer, 5′-GGGTTTTGTGTTCAATCTTATGATTACT-3′; *SmSept7.1* reverse primer, 5′- GATGGACCAGGATAATCAGTGTTG-3′, *SmSept7.2* forward primer 5′-CGCGTTTCGATGATTACATATCTG-3′; *SmSept7.2* reverse primer 5′- GGAGCAATAAAGTAAATGCATGCA - 3′. Efficiency of the PCR for each pair of septin specific primers was estimated by titration analysis to be 100%±5 [36] (not shown).The qPCRs were performed in triplicate followed an initial denaturation at 95°C for 3 min and 40 cycles of 30 sec at 95°C and 30 sec of 55°C. The specificity of the PCR product was verified by a melting curve: 1 min at 95°C, 1 min at 55°C and a ramp from 55 to 95°C with an increasing rate of 1°C/min. Absolute quantification was undertaken using copy number standards, i.e. 10-fold serial dilutions of each septin clone. Copy number of each clone dilution was calculated through the relationship between the molecular mass of the clone and the Avogadro constant. Absolute copy number of septin transcripts was estimated by interpolation of the sample PCR signals from a standard curve [36]. Biological replicates were performed. In addition, relative quantification was undertaken in order to evaluate the expression of the four septin genes within developmental stages of *S. mansoni*. *S. mansoni* glyceraldehyde 3-phosphate dehydrogenase (*Sm*GAPDH; GenBank M92359), forward primer, 5′-TGTGAAAGAGATCCAGCAAAC-3′; reverse primer, 5′-GATATTACCTGAGCTTTATCAATGG-3′ was employed as a reference gene, with these PCRs carried out as above. The *E* ^−ΔCt^ method, a variation of the Livak method that incorporates the amplification efficiency values (E) for each pair of primers, was employed to determine the expression of septins relative to *Sm*GAPDH, within each sample, i.e. each developmental stage analyzed [37]. Bioinformatics analyses were performed using RNA-seq reads from libraries of adult worms and of cercariae [32]. A tally of the RNA-seq reads aligning to the transcripts encoding the four septins was compiled based on outcomes of a blastn search, to assess relative abundance of each septin.

### Confocal imaging

Developmental stages (miracidium, sporocyst, cercaria and schistosomulum) of *S. mansoni* were dispensed in tissue culture medium into cell culture inserts incorporating polyethylene terephthalate track-etched membranes with a pore size of 8 µm (BD Falcon, BD Biosciences, Durham NC), mounted in wells of plastic 24-well tissue culture plates. Schistosomes were fixed in 4% paraformaldehyde (PFA) by diluting 16% PFA (EMS, Electron Microscopy Sciences) in 1× phosphate-buffered saline (PBS) for 1 h at 4°C. Subsequent steps were performed on a laboratory shaker. Worms were permeabilized with Triton X-100 in PBS (0.2%) for 60 min at 25°C, followed by three washes in PBS with 0.05% of Tween-20 (PBS-T). The blocking step was carried out overnight at 4°C in PBS containing 5% normal goat serum (NGS), followed by incubation with the primary antibody (1∶50 dilution) for 2 d at 4°C. Samples were washed three times with PBS-T and incubated in 5% NGS for 20 min at 25°C as a second blocking step. Anti-mouse IgG conjugated to Alexa Fluor 633 Goat (Invitrogen) was added to the blocking solution to a final dilution of 1∶300, after which samples were incubated in the dark for 90 min at 37°C. The samples were subsequently stained for 30 min at 25°C with 4′,6-diamidino-2-phenylindole (DAPI) at 300 nM and Alexa Fluor 568 phalloidin (Invitrogen) at 165 nM in PBS containing 1% bovine serum albumin (BSA) for 30 min at 25°C. After samples were air dried, they were mounted in Fluoromount-G (EMS) on glass slides.

Confocal images were obtained using a Carl Zeiss LSM 710 system, which includes a Zeiss Axio Examiner Z1 microscope and a Quasar 32-channel spectral detector. Samples were scanned sequentially using a Plan-Apochromat 63×/1.40 Oil DIC objective. For acquisition of signals from the DAPI channel, targets were excited with a 405 diode laser line and emission was filtered in a band between 410 and 585 nm. Immunolabeling (Alexa Fluor 633) was revealed by excitation with a diode 633 laser line, with emission recorded between 638–747 nm. Phalloidin labeling of actin filaments was excited with a 561 diode laser and emission recorded from 572 to 630 nm. Optical confocal sections were generated by adjusting the pinhole to one Airy unit using the most red-shifted channel, producing an optical section of ∼0.7 µm in all channels. Confocal images were captured in sequential acquisition mode to avoid excitation bleed-through, particularly apparent with DAPI. Image frames measured 1024×1024 pixels with a pixel dimension of 0.132 µm. Images manipulation was undertaken with the assistance of Zen 2009 software (Carl Zeiss). Manipulations were limited to adjustment of brightness, cropping, insertion of scale bars and the like; image enhancement algorithms were applied in linear fashion across the image and not to selected aspects. Control images were adjusted similarly.

## Results

### *Schistosomes* displays four discrete septins

Four genes, Smp_041060, Smp_060070, Smp_003620 and Smp_029890, encoding putative septins were identified in the *S. mansoni* genome by interrogating the database in a tBLASTn search with all described human septin protein sequences as queries. Eventual discrepancies between gene predictions and actual transcript data were assessed. Utilizing the database of *S. mansoni* ESTs for a BLASTn search with each of four newly predicted *S. mansoni* septins, we observed that Smp_041060 included 5′ residues that did not correspond entirely with any EST and ESTs AM042809 and AM043866 exhibited a different 5′ terminus. These sequences were aligned to assemble a putative sequence for this transcript. Confirmation of the existence of this transcript was investigated by reverse transcription PCR utilizing primers flanking the full-length coding sequence (CDS) of the putative gene, followed by nucleotide sequencing. Sequences of the other three predicted transcripts were similarly confirmed. The sequences of the full-length CDS of *Sm*SEPT5, *Sm*SEPT7.1, *Sm*SEPT7.2 and *Sm*SEPT10 have been assigned GenBank accessions KC916723, KC916724, KC916725 and KC916726, respectively.

Sequence alignment of the four polypeptides predicted to be encoded by these transcripts using BLASTp with the 13 human septins as a database indicated that two of the *S. mansoni* septins, Smp_003620 and Smp_060070, displayed higher identity with the human septin SEPT7 (47% and 55% identity, respectively). Hence we termed the putative proteins *Sm*SEPT7.1 and *Sm*SEPT7.2. The other *S. mansoni* septins, Smp_029890 and Smp_041060, displayed higher identities with human septin 10 (65% identity) and septin 5 (57% identity); there have been named *Sm*SEPT10 and *Sm*SEPT5, respectively. The four schistosome septins have predicted molecular masses of 48–58 kDa and display hallmarks of the septin family: a highly conserved GTPase domain containing the G1 (GXXXXGKS/T), G3 (DXXG) and G4 (XKXD) motifs [38], a septin unique region [39], a variable N-terminus, and a C-terminal region predicted to form a coiled coil structure (COILS program [40]) (Figure 1).

A phylogenetic tree established using Bayesian inference of a multiple alignment of the conserved GTPase domains of the four *S. mansoni* septins with septins from selected deuterostomes and protostomes revealed that the orthologous *S. mansoni* septins *Sm*SEPT 5, *Sm*SEPT10, and *Sm*SEPT 7.1 with *Sm*SEPT 7.2 clustered in the groups SEPT2, SEPT6 and SEPT7, respectively, with strong statistical support (Figure 2). Proteins from these groups comprise a known human hetero-oligomeric septin complex [18]. Examination of the phylogenetic tree presented in Figure 2 indicated, with high posterior probability, that all the septin groups are monophyletic and their branches have an origin in the center of the tree, predicting that divergence of all septin groups preceded the protostome-deuterostome split. It is noteworthy that schistosomes, like the other protostomes sampled, lacked septins of the SEPT3 group, suggesting that this gene was lost early in this branch of evolution. The phylogram also indicated with strong statistical support that deuterostome genes encoding groups SEPT7 and SEPT6 form a monophyletic branch. This suggested that only a single copy from each of these families was present in the last common ancestor of deuterostomes and protostomes. Gene duplications that resulted in several copies of septins from group SEPT6 in humans likely occurred after the divergence of the two lineages. A peculiar scenario was evident in the SEPT2 group, in which septins of protostomes and deuterostomes did not segregate in the branch structure. This suggests that at least one event of gene duplication in this family preceded the divergence between species analyzed here. The only gene duplication observed among the four new schistosome septins was in the SEPT7 group. Curiously, a similar duplication did not occur in the orthologous human group, where only one form of SEPT7 is known [4].

Access to genome sequences of several other species of the phylum Platyhelminthes [41] facilitated analysis of putative septin sequences among flatworms at large. Phylogenetic analyses of the tapeworms *Echinococcus multilocularis, E. granulosus, Taenia solium* and *Hymenolepis microstoma* revealed the presence of four septin genes that cluster into the same groups of schistosome septins (Figure S1), indicative of a conservation of septin structures among trematodes and cestodes.

### Coordinated expression of septins among developmental stages of *S. mansoni*

**Figure 3:**
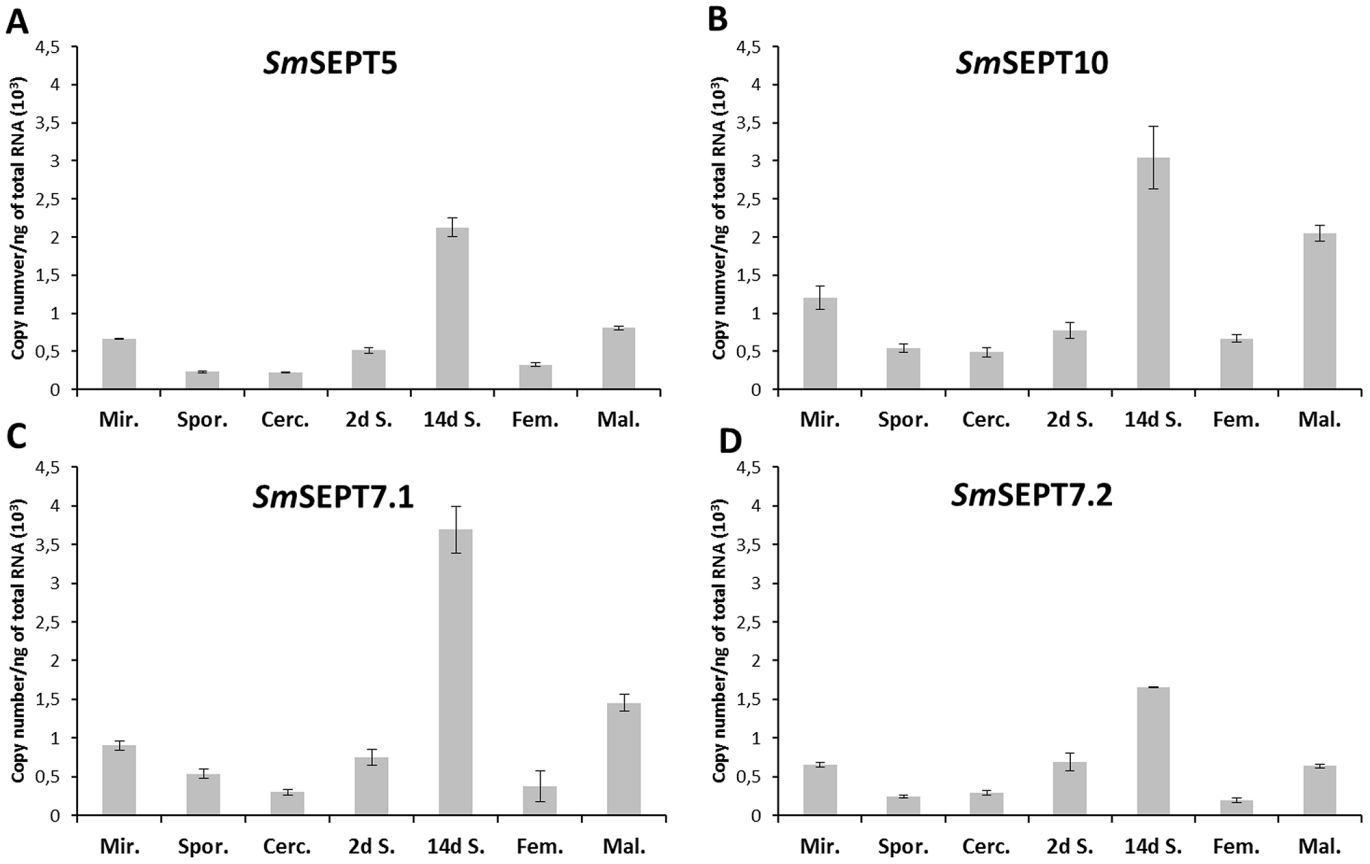
Expression profiles of septins among developmental stages of *Schistosoma mansoni*. Expression analysis by qPCR of each of four septins in seven developmental stages of the blood fluke. Panels A to D represent expression levels of *SmSept5*, *SmSept10*, *SmSept7.1* and *SmSept7.2*, respectively. Absolute quantification was used to evaluate the expression levels and is presented as copy number per ng of total RNA. Copy number of each transcript was estimated by interpolation of the sample signal from a standard curve for each gene; bars represent standard deviation of the mean of three technical replicates. Mir, miracidia; Spor, sporocyst; Cerc, cercariae; 2 d S, 2 days old schistosomula; 14 d S, 14 day old schistosomula; Fem, adult female; Mal, adult male. (A biological replicate revealed the same trend among stages; Figure S2.)

The expression profile of septins in developmental stages of the *S. mansoni* was investigated by quantitative PCR. At the outset, absolute quantification was employed to normalize septin expression among different developmental stages [42]–[45]. (Gene expression analysis based on normalization to a reference gene, by contrast, may be challenging in the absence of accurate information on the reference gene expression throughout the developmental stages analyzed in the present study.) The expression profile of the four septin genes exhibited similar trends among the developmental stages (Figure 3). This outcome was confirmed in a biological replicate (Figure S2).Given the propensity of septins to form hetero-filaments [18], this coordinated expression of all septin groups suggested that functional filaments of septins in schistosomes may be composed of multiple septin proteins. In order to further investigate the coordinated expression of the four septin genes, relative quantification was undertaken using *Sm*GAPDH as a reference gene. The relative expression levels of the four septins were detected within each individual stage. In concordance to the findings with absolute quantification PCR (Figure 3), the relative abundance of the four septin transcripts was similar among the stages studied (Figure S3). Additionally, the relative contribution of each septin gene in the adults and cercarial stages of *S. mansoni*, was assessed by interrogation of publicly available RNA-seq data for these two stages [32]; a similar pattern of transcript abundance was apparent (Figure S4). Together, analyses by absolute and relative qPCR and of RNA-seq libraries [32] indicated that schistosome septins exhibit coordinated expression during the development cycle of the parasite.

### Ubiquitous expression in the schistosomulum

Immunolocalization analyses were undertaken using affinity purified antibodies raised against two of the four *S. mansoni* septins, *Sm*SEPT5 and *Sm*SEPT10. At the outset, western blots were performed with two recombinant schistosome septins and lysates of adult schistosomes to ascertain the specificity of the antibodies. Minimal cross-reactivity was apparent even to excessive quantities of septins. Anti-*Sm*SEPT5 immunoglobulin recognized *Sm*SEPT5 strongly and *Sm*SEPT10 weakly. In similar fashion, anti-*Sm*SEPT10 immunoglobulin recognized *Sm*SEPT10 strongly but only weakly recognized *Sm*SEPT5 (Figure S5, panels A, C). Moreover, only single bands reacted in soluble lysates of adult schistosomes, incubated with anti-*Sm*SEPT5 or anti-*Sm*SEPT10 (Figure S5, panels B, D), indicating negligible cross-reactivity at physiological concentrations of the targets. The same trend of expression was revealed by the imaging analysis regardless of the antibody used - anti-*Sm*SEPT5 or anti-*Sm*SEPT10, consistent with the proposition that schistosome septins form hetero-oligomeric complexes. Representative images labeled with anti-*Sm*SEPT5 and anti-*Sm*SEPT10 in two developmental stages are presented in the Figures S6 and S7 to illustrate the similarity of the localization profiles. Images from samples incubated in the secondary antibody only showed minimal, though marginally detectable, signals in the septin channel (Figure S8).

**Figure 4:**
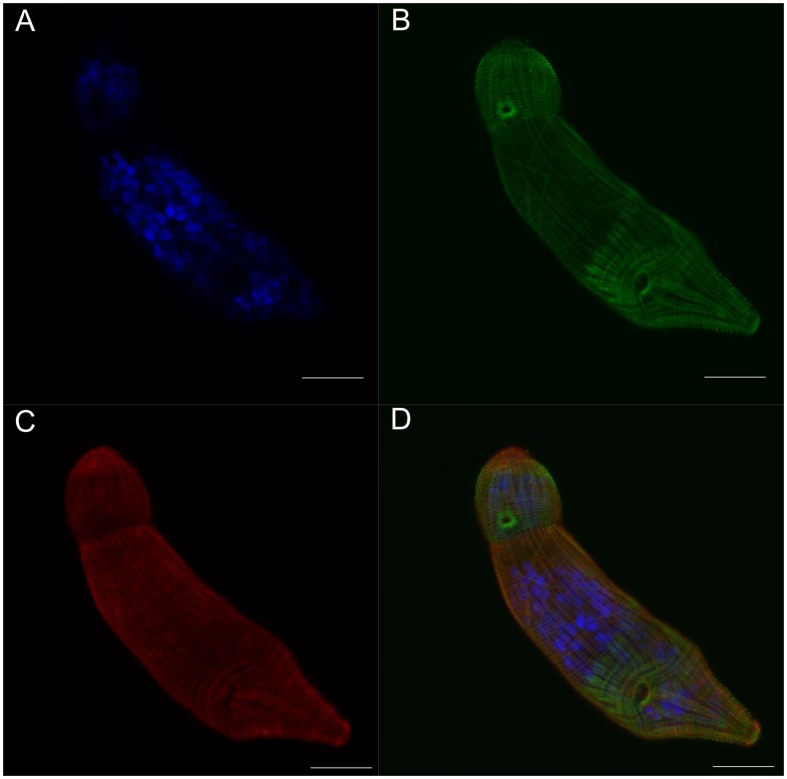
Septin fibers co-localize with actin fibers at the surface of the schistosomulum. Panel A: Cross-section at the surface of a two day old schistosomulum showing DAPI stained nuclei. B: F-actin structure stained with phalloidin. C: Septin labeled with anti-*Sm*SEPT5. D: Merged channels. Scale bars, 20 µm.

**Figure 5:**
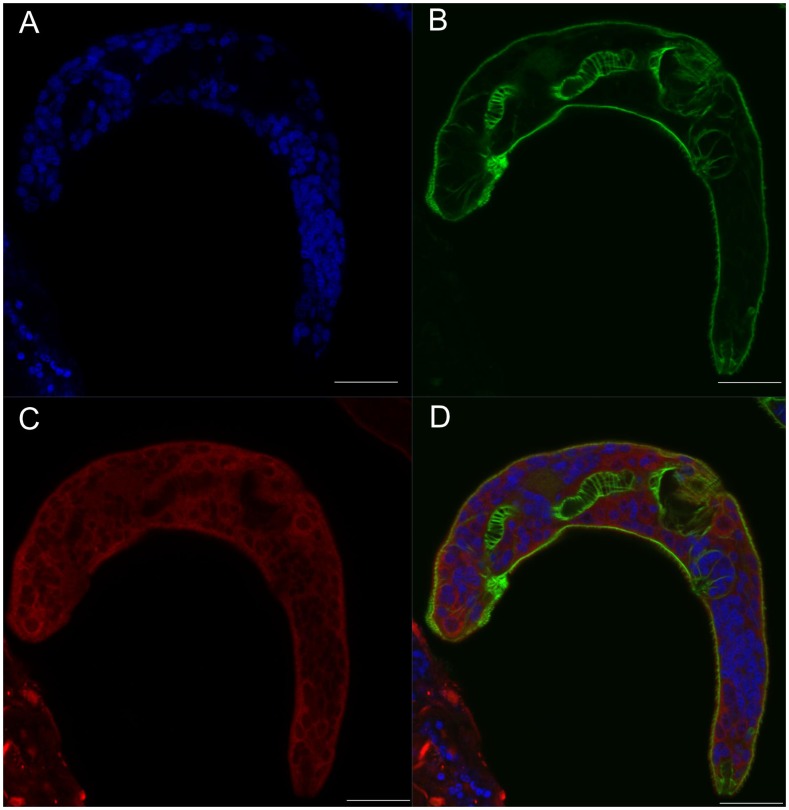
Septins are ubiquitous in tissues of the schistosomulum. Cross section at an inner intersection of a schistosomulum cultured for 14 days. Panel A: Nuclei stained with DAPI. B: F-actin structure stained with phalloidin. C: Septin labeled with anti-*Sm*SEPT5. D: Merged channels. Scale bar, 20 µm.

**Figure 6:**
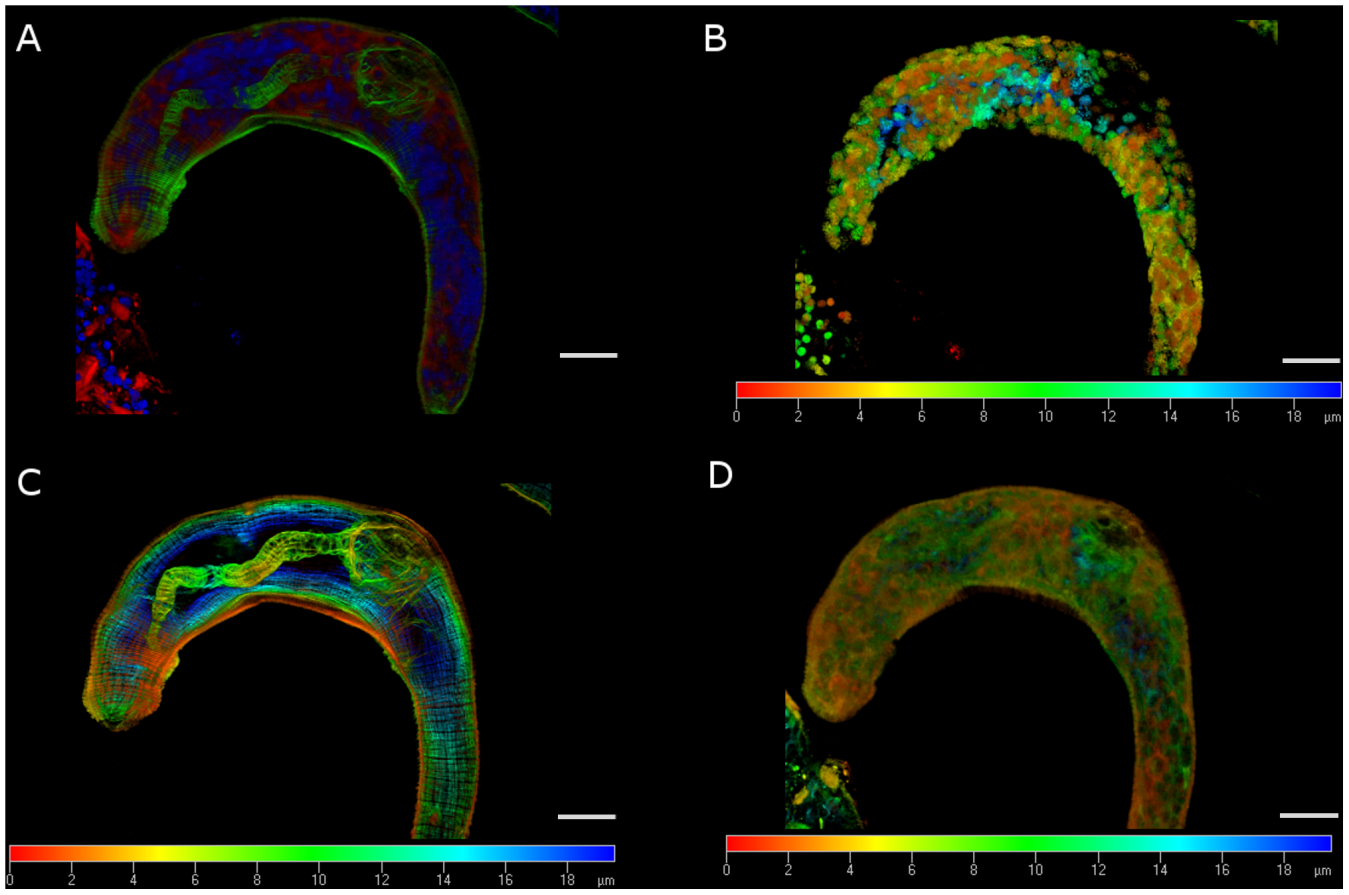
Septins are ubiquitously expressed in the schistosomulum. Projections of 40 optical sections at 0.5 µm intervals of a schistosomulum cultured for 14 days. (A) Projection of DAPI stained nuclei (blue), phalloidin stained F-actin (green), and septin labeled with anti-*Sm*SEPT5 immunoglobulin (red). (B) Individual projection of nuclei stained with DAPI. (C) Projection of phalloidin staining several layers of F-actin throughout the schistosomulum. (D) Individual projection of septin staining with anti-*Sm*SEPT5. The color scale bar represents the depth; white scale bars, the length −20 µm.

In parallel to the immunolocalization with anti-septin antibodies, filaments of schistosome actin were labeled with phalloidin, a ligand of fungal origin that binds actin polymers. Two- and 14-day-old schistosomula exhibited similar immunofluorescence profiles (Movies S1, S2). Septin fibers could be identified at the worm surface, along with actin fibers (Figure 4). Septins were also evident in deeper layers, usually at the periphery of the cells (Figure 5). Three-dimensional renderings of confocal images including signals from the DAPI, phalloidin and septin probes revealed ubiquitous expression of septins (Figure 6). By contrast, actin was more prominent in muscle layers and the gut (Figure 6B).

### Septins localize in superficial structures and germ cells of the miracidium and sporocyst

**Figure 7:**
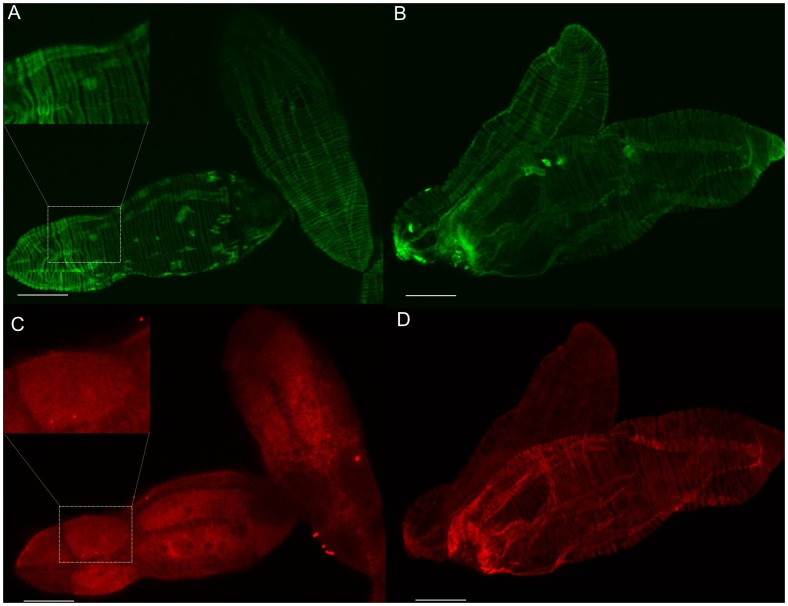
Superficial structures of miracidia and sporocysts of *Schistosoma mansoni*. Panels A and C represent a superficial cross section of miracidia and B and D depict a superficial layer of two-day-old sporocysts. The green panels A and B show F-actin stained with phalloidin conjugated with Alexa Fluor 568. The red panels C and D reveal septin structures labeled with anti-*Sm*SEPT10 immunoglobulin. The upper left inset in panel C highlights an epidermal plate of a miracidium, rich in septins, and in panel A the same region revealed a muscular structure stained with phalloidin. Scale bar, 20 µm.

**Figure 8:**
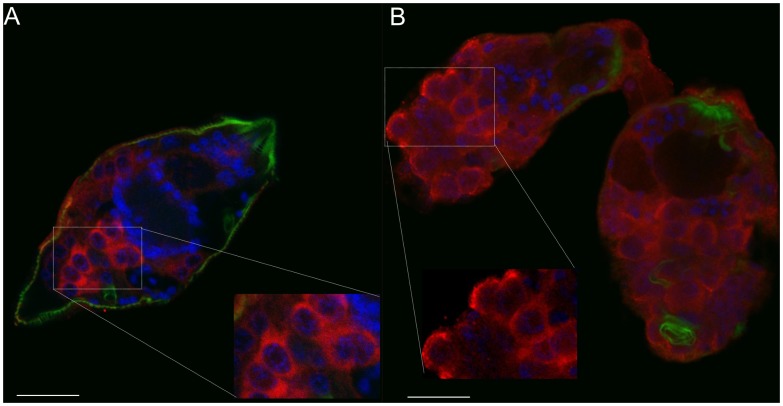
Septin in germ cells of miracidia and sporocysts of schistosomes. Confocal optical sections of a miracidium (panel A) and a two day old sporocyst (B); nuclei stained with DAPI (blue) and actin filaments stained with phalloidin conjugated with Alexa Fluor 568 (green). Probing with anti-*Sm*SEPT10 immunoglobulin (red) revealed the prevalence of septin in germ cells of both miracidia and sporocysts. The insets of A and B highlight germ cell rich regions in these developmental stages. Scale bar, 20 µm.

Miracidia and sporocysts cultured for two days were permeabilized and probed with anti-septin immunoglobulins, followed by incubation with an Alexa Fluor 633-conjugated secondary antibody. Confocal imaging allowed the sampling of increasingly deeper (internal) layers of these stages of the blood fluke which, in turn, precisely localized septins in organs and tissues (Movie S3; Figures S9, S10). The superficial layers of the miracidium expressed septins on the epidermal plates (Figure 7), which contain the cilia of the motile larva. Images of superficial layers of the sporocyst revealed colocalization of actin and septin in the longitudinal and circular muscle layers (Figure 7D). Moreover, septins were prominent in optical sections of germs cell in miracidia (Figure 8A) and two-day-old sporocysts (Figure 8B). Colocalization of septin and actin was observed in the superficial optical sections of these larval stages, though not in deeper sites.

### Protonephridial ducts of the cercaria are septin rich structures

**Figure 9:**
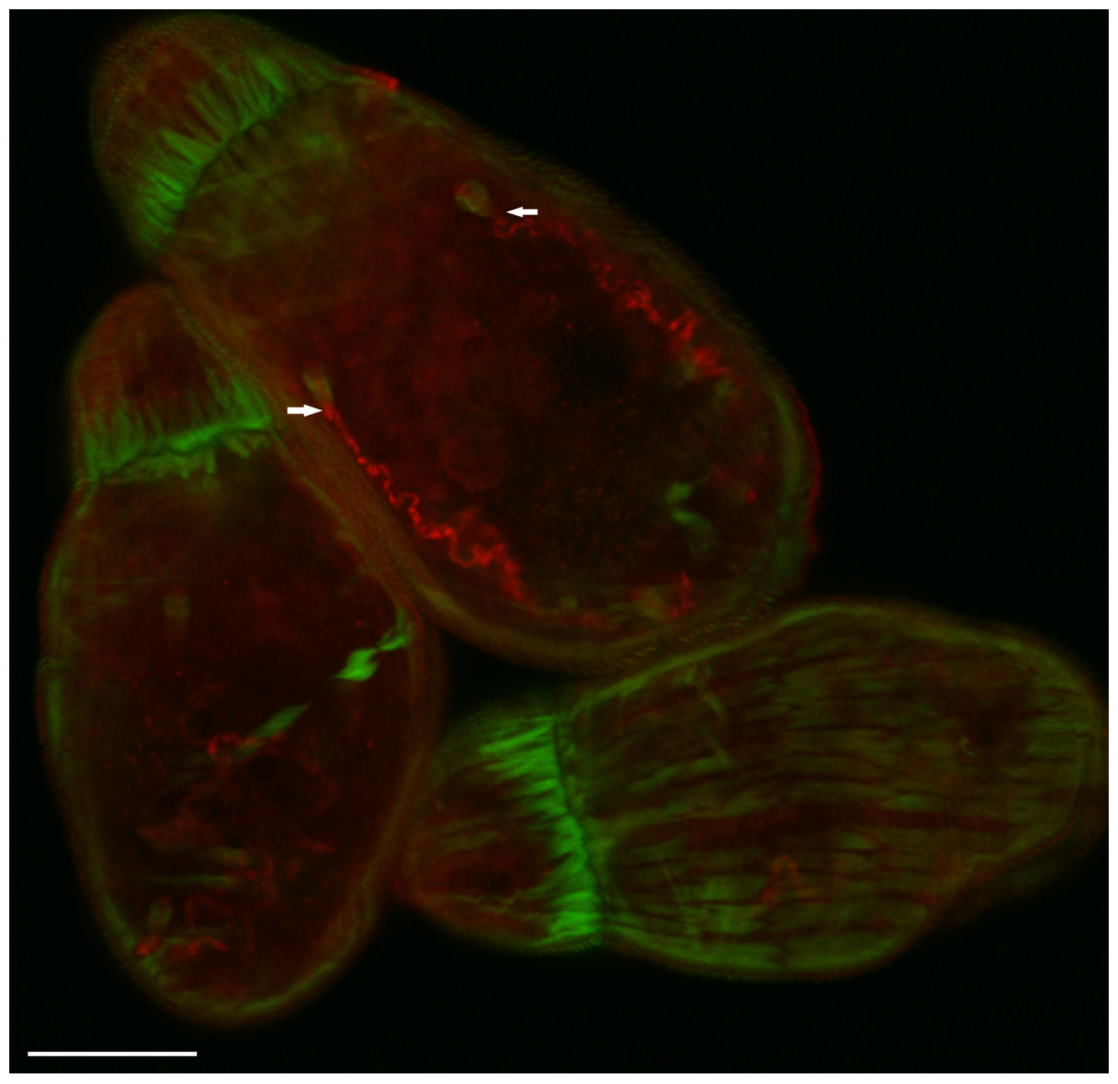
Protonephridial ducts of the schistosome cercaria are septin rich structures. Optical section of cercariae labeled with phalloidin (green) and anti-*Sm*SEPT10 (red). The arrows indicate the flame cells at anterior termini of protonephridial canals. Scale bar, 20 µm.

Robust staining of septin was seen in the cercaria along the protonephridial ducts that extend laterally down both sides of the larva; characteristic flame cells are located at the termini of the canals (Figure 9, arrows; Movie S4). The protonephridium consists of a network of osmoregulatory tubules comprising the excretory system of the larva and extends nearly the entire length of the body [46], [47]. Septins appear to occur at the collecting tubules of this osmoregulatory system (Figure 9); the role of septins in these structures remains to be elucidated.

## Discussion

Septins are cytoskeleton components formed from hetero-oligomeric complexes which assemble into higher-order structures such as filaments and rings. Whereas septins are evolutionarily conserved and widely distributed among eukaryotes, until now septins have not been reported in schistosomes or indeed in any flatworm. Four schistosome septins are reported here; they are members of three discrete septin groups, with a duplication verified in SEPT7 group. A similar gene organization was apparent in the genomes of four cyclophyllidean tapeworms including *E. multilocularis*. The conserved gene organization between trematodes and cestodes indicates an essential role of these proteins across the phylum.

The flukes and tapeworms investigated displayed members of the same three septin groups described previously in some other invertebrates, including *Drosophila melanogaster* and *Caenorhabditis elegans*. The SEPT3 group appears to be absent from these taxa [20], [48], suggesting that a deletion occurred in a common ancestor. Despite the absence of one of the groups, the functional assembly of septins into heter-oligomeric structures is maintained [49], [50]. Phylogenetic analysis involving nine metazoans [20], including the sea urchin *Strongylocentrotus purpuratus* and the sea squirt *Ciona intestinalis* which, as representatives of the echinoderms and non-vertebrate chordates, respectively, occupy informative phylogenetic positions with respect to the evolution of mammals, revealed that they share orthologues of all human septin groups. This suggests that these four septins groups evolved before the appearance of the vertebrates and supports the hypothesis of a deletion event in some lineages of the invertebrates. Moreover, schistosomes exhibited only one isoform for groups SEPT2 and SEPT6 in contrast to the situation in humans where several isoforms of the groups occur. Septin configuration in schistosomes may resemble an ancestral arrangement, whereas successive duplications and mutations have endowed the human isoforms with a more specialized role(s).

Real-time PCR analyses indicated that transcription of genes encoding the four schistosome septin was maintained at approximately the same ratio throughout the developmental cycle. This suggested that a septin heterocomplex might exhibit a similar composition in the diverse morphological stages of the schistosome, although deeper investigation will be required to precisely define the proportion of the different group members of the septin heterocomplex in *S. mansoni*. Confocal imaging revealed that muscle layers were rich in actin and septin, suggesting cell specific co-expression of these cytoskeleton elements. Their co-localization was more evident in the sporocyst, which undergoes shedding of ciliated epidermal plates and the emergence of a new tegument during infection of the snail [51]. Co-localization of actin and septin is well-known in mammals [52], [53]. Rapid-freeze, deep-etch immuno-replica electron micrographs reveal associations of septins with actin-based membrane skeletons of kidney cells [54]. Septins can be recruited to an actin bundle through the interaction with the adaptor protein anillin of *Xenopus laevi*s [53]. BLASTp searches using *X. laevis* anillin allowed the identification of a homologue from *S. mansoni* (XP_002576415.1; 43% similar over 177 positions). Analysis using the CDD tool at NCBI (not shown) revealed an anillin pleckstrin homology (PH) domain (cd01263) in the schistosome orthologue, suggesting that it performs a similar role.

Confocal immunolocalization micrographs revealed septins in the ciliated epidermal plates of the miracidium. Septins are required for ciliogenesis and constitute a diffusion barrier at the base of the ciliary membrane in mammalian cells and *Xenopus* embryos [49], [55]. The cilium of the miracidium exhibits microtubules with a 9+2 pattern [56], typical of eukaryotic motile cilia [57]. Since the cilium is a highly conserved organelle in eukaryotes [57], septins in the epidermal plates of the miracidium likely display a similar organization to the septins found in cilia of mammalian cells, and may perform a similar role. The prominent expression of septins in germ cells of both the miracidium and the mother sporocyst and absence of phalloidin staining in this tissue together indicated that actin and septins of schistosomes do not always act in concert. In like fashion, earlier reports indicate that actin was not detectable in germ cells when miracidia and sporocysts were probed with phalloidin [47], [58], [59]. A pattern of staining for β-tubulin in the germ cells has been reported [48] that is similar to the localization of septins in germ cells described here, which together suggests a cellular co-expression of these filaments as well. Associations of septins and microtubules are well known [60]–[63]. In *D. melanogaster*, Peanut, SEPT1 and SEPT2 have been identified in male germ cells [64]. Likewise, septins are known from the mammalian germ cells. Sept4 null male mice are sterile due to immotile spermatozoa with defective annulus [65], [66], and diminished expression of SEPT12 transcripts is evident in the testicles of infertile men [65]. Moreover, septin 12 plays a key role in terminal differentiation of germ cells in both humans and mice [67]. Septins are best known for their role in cytokinesis [9]–[14] and we speculate that the role of septins in miracidial and sporocyst germ cells might be related to the mitotic activity of these cells.

Schistosomula cultured for two or 14 days showed ubiquitous localization of septin in contrast to a more restricted distribution in the other stages. Ubiquitous septin localization has also been reported for some human septins [68]; it is feasible that septins are involved in some similar functions in human and schistosome cells and tissues. To conclude, this is the first description of septins of a schistosome or any platyhelminth. Four septins were identified and they were differentially expressed among developmental stages of the blood fluke. Confocal imaging indicated that schistosome septins undertake specialized roles in this pathogen. Detailed evaluation of schistosome septins can be expected to clarify the relationship of this category of proteins with cellular and physiological functions and to deliver deeper understanding of schistosome physiology and anatomy and its roles in the host-parasite relationship.

## Supporting Information

Figure S1 **Phylogenetic analysis of septins of flatworms.** Phylogenetic tree (Bayesian inference) generated from the multiple alignment of the conserved GTPase domains of septins of the trematodes *Schistosoma mansoni, S. haematobium, S. japonicum* and the cestodes *Echinococcus multilocularis, E. granulosus, Taenia solium* and *Hymenolepis microstoma*. The numbers on the tree nodes are posterior probabilities calculated by MrBayes. Branches with the four discrete groups of septins are enclosed by the dotted lines. Species are identified by the small circles of different shapes and colors as indicated in the lower panel.
(TIF)

Figure S2 **Biological replicate of the expression profiles of septins among developmental stages of** ***Schistosoma mansoni*** **.** Expression analysis by qPCR of each of four *S. mansoni* septins in seven stages of the blood fluke; panels A to D present expression levels of *SmSept5*, *SmSept10*, *SmSept7.1* and *SmSept7.2*, respectively. Absolute quantification was used to evaluate the expression levels and is presented as copy number per ng of total RNA. Copy number of each transcript was estimated by interpolation of the sample signal from a standard curve for each gene; error bars represent standard deviation of the mean of three technical replicates. Mir, miracidia; Spor, sporocyst; Cerc, cercariae; 2 d S, 2 days old schistosomula; 14 d S, 14 day old schistosomula; Fem, adult female; Mal, adult male.
(TIF)

Figure S3 **Septins display similar transcription patterns in developmental stages of** ***Schistosoma mansoni*** **.** Relative quantification qPCRs using SmGAPDH as reference gene were performed to assess the relative expression of the four septin transcripts within different life stages of *S. mansoni*: adult male worms, adult female worms, schistosomula cultured for 14 days, cercariae and miracidia displayed a similar pattern of expression for the four septins. Expression of each gene relative to SmGAPDH is presented as relative abundance of each septin transcript considering the lowest value equal to 1.
(TIF)

Figure S4 **Bioinformatics analysis confirms that septin genes from adult worms and cercariae exhibit similar patterns of transcription.** A blastn search in RNA-seq reads from libraries of cercariae (panel A) and mixed sex, adult worms (panel B) reported by Protasio and coworkers [32] was performed and a tally of the RNA-seq reads aligning to the four transcripts encoding septins was compared. Values are expressed as number of aligned reads per million of reads per kilobase of transcript. Data for cercariae represent the average from three independent libraries whereas adult worm data were from a single experiment. This analysis revealed a pattern of expression among the four septin genes very similar to that ascertained by the relative qPCR.
(TIF)

Figure S5 **Western blot analysis of** ***S. mansoni*** **septin antibodies.** Panel A: Anti-*Sm*SEPT5 recognizes recombinant *Sm*SEPT5 (0.5 µM) at 53.8 kDa (left lane) and weakly recognizes *Sm*SEPT10 (0.5 µM) at 50.5 kDa (left lane). B: Anti-*Sm*SEPT5 recognizes a single band in a lysate of mixed sex adults, at the expected molecular mass. C: Anti-*Sm*SEPT10 recognizes recombinant *Sm*SEPT10 (0.5 µM) (right lane) and weakly recognizes *Sm*SEPT5 (left lane). D: Anti-SmSEPT10 recognizes a single band in a lysate of mixed sex adults, at the expected molecular mass. Molecular size standards in kilodaltons (kDa) are shown at the left of the blots.
(TIF)

Figure S6 **Staining of a miracidium with anti-** ***Sm*** **SEPT5 and anti-** ***Sm*** **SEPT10 immunoglobins.** Confocal optical sections of a miracidium labeled with *Sm*SEPT5 (panel A) and anti-*Sm*SEPT10 (B) showing a similar pattern of localization irrespective of which anti-septin probe was deployed. Nuclei stained with DAPI (blue) and actin filaments stained with phalloidin conjugated with Alexa Fluor 568 (green). Probing with both antibodies revealed the prevalence of septin in germ cells of miracidia. Scale bar, 20 µm.
(TIFF)

Figure S7 **Staining of schistosomula with anti-** ***Sm*** **SEPT5 and -** ***Sm*** **SEPT10 immunoglobulins.** Confocal optical sections of schistosomula cultured for 14 days labeled with *Sm*SEPT5 (panel A) or anti-*Sm*SEPT10 (B) immunoglobulins revealed ubiquitous septin localization in this stage for both probes. Nuclei stained with DAPI (blue) and actin filaments stained with phalloidin conjugated with Alexa Fluor 568 (green). Scale bar, 20 µm.
(TIFF)

Figure S8 **Control samples incubated with secondary antibody.** Panel A: F-actin structure stained with phalloidin. B: Miracidia stained only with the secondary antibody conjugated to Alexa Fluor 633. Other developmental stages presented similar background level signals (not shown).
(TIF)

Figure S9 **Septins are expressed in the miracidium.** Serial optical sections (×28) at 0.5 µm intervals, projected at the z-axis using the Zen software. Panel A: Projections of DAPI (blue), phalloidin (green) and anti-*Sm*SEPT5 (red) signals. B: Individual projection of nuclei stained with DAPI. C: Projection of phalloidin staining several layers of F-actin throughout the miracidium. D: Individual projection of septin labeled with anti-*Sm*SEPT10. The color scale bar represents the depth, and white scale bars represent the length, 20 µm.
(TIF)

Figure S10 **Septins are expressed in the sporocyst.** Projection of 28 optical sections of sporocysts cultured for two days. Panel A represents projections of nuclei (blue), actin (green) and septin (red). B: Individual projection of nuclei stained with DAPI. C: Projection of phalloidin staining several layers of F-actin throughout the sporocyst. D: Individual projection of septin labeled with anti-*Sm*SEPT10. The color scale bar represents the depth. White scale bars, the length, 20 µm.
(TIF)

Movie S1 **Serial optical section of two day old schistosomula.** Series of 32 optical sections of two day old schistosomula at intervals of 0.5 µm. The blue, green and red channels revealed staining with DAPI, actin, and septin, respectively. The fourth panel presents a merged image of the three channels.
(MP4)

Movie S2 **Serial optical sections of 14 day old schistosomula.** Series of 43 optical sections of 14 day old schistosomula at intervals of 0.5 µm. The blue, green and red channels revealed staining with DAPI, actin, and septin, respectively. The fourth panel presents a merged image of the three channels.
(MP4)

Movie S3 **Serial optical sections of miracidia.** Series of 29 optical sections of miracidia at intervals of 0.5 µm. The blue, green and red channels revealed staining with DAPI, actin, and septin, respectively. The fourth panel presents a merged image of the three channels.
(MP4)

Movie S4 **Serial optical sections of cercariae.** Series of 32 optical sections of cercariae at 0.8 µm intervals. The blue, green and red channels revealed staining with DAPI, actin, and septin, respectively. The fourth panel displays the merged image of the three channels scanned.
(MP4)
