# Supplementary figures and images for: Septins of Platyhelminths: Identification, Phylogeny, Expression and Localization among Developmental Stages of Schistosoma mansoni
Source: PLoS Negl Trop Dis. 2013 Dec 19;7(12):e2602. doi: 10.1371/journal.pntd.0002602 (PMC3868516; doi:10.1371/journal.pntd.0002602)

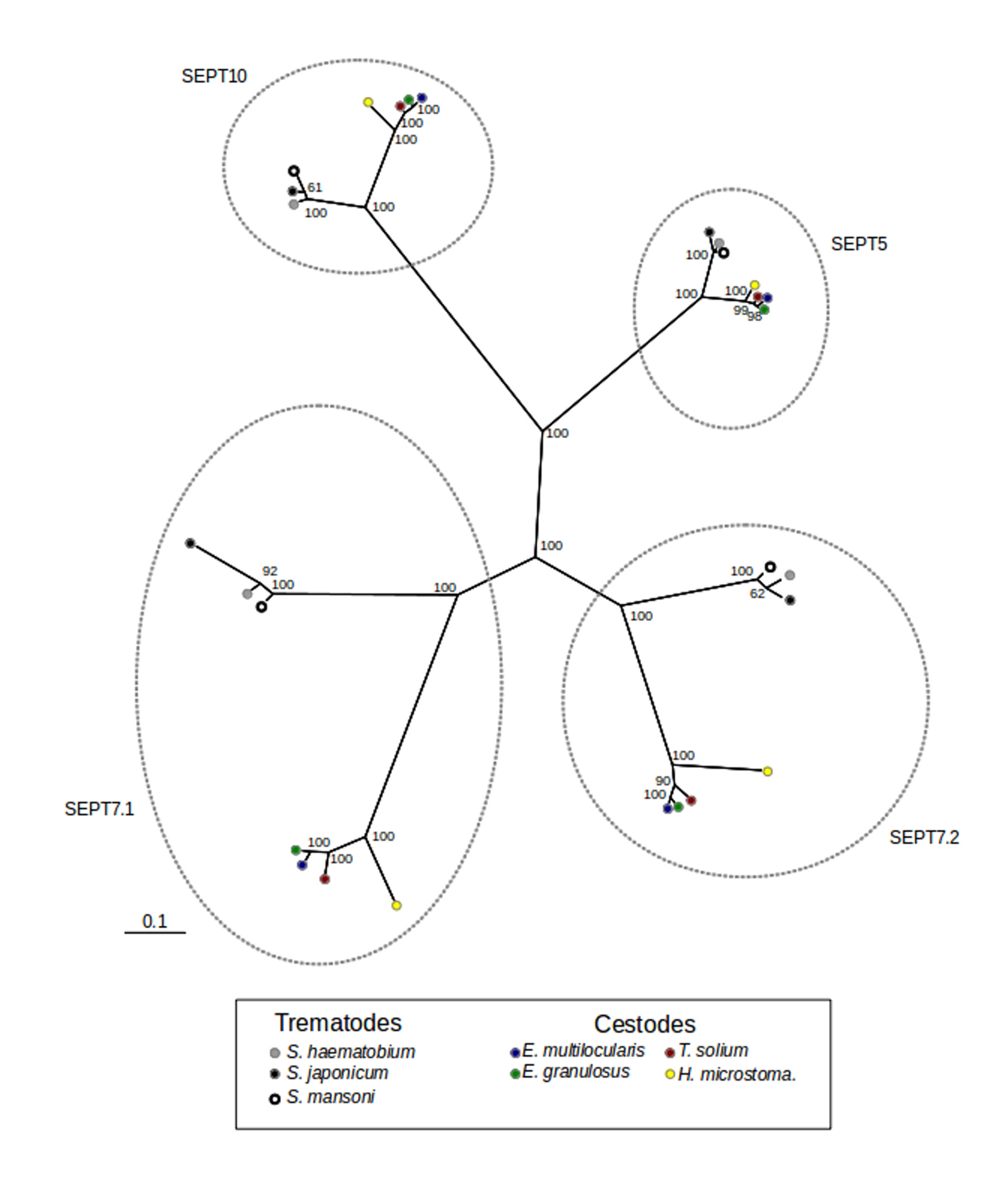

Supplement: Figure S1 — Phylogenetic analysis of septins of flatworms. Phylogenetic tree (Bayesian inference) generated from the multiple alignment of the conserved GTPase domains of septins of the trematodes Schistosoma mansoni, S. haematobium, S. japonicum and the cestodes Echinococcus multilocularis, E. granulosus, Taenia solium and Hymenolepis microstoma. The numbers on the tree nodes are posterior probabilities calculated by MrBayes. Branches with the four discrete groups of septins are enclosed by the dotted lines. Species are identified by the small circles of different shapes and colors as indicated in the lower panel. (TIF) [file pntd.0002602.s001.tif]

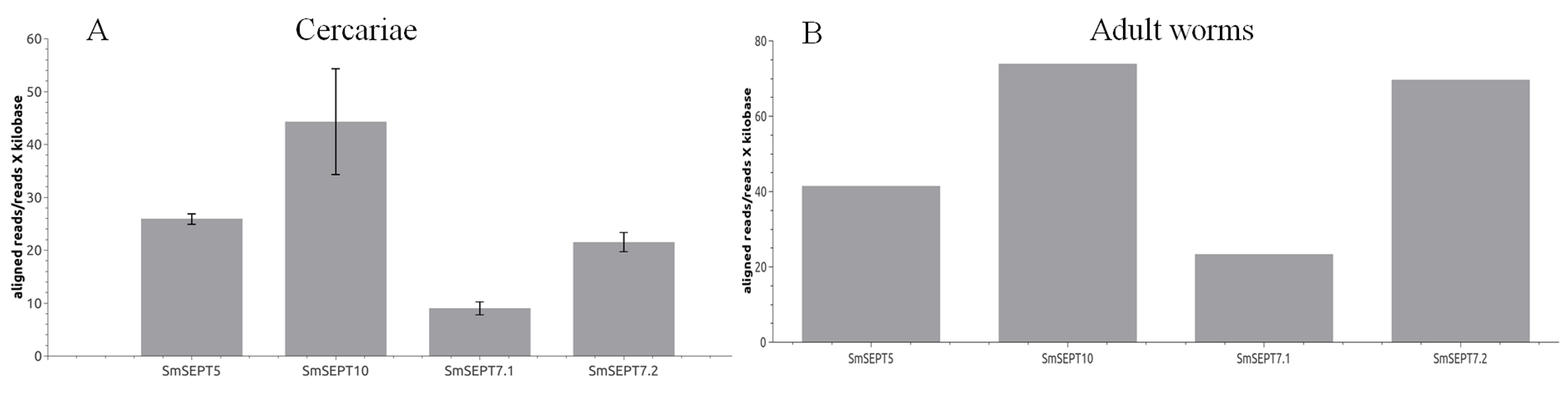

Supplement: Figure S4 — Bioinformatics analysis confirms that septin genes from adult worms and cercariae exhibit similar patterns of transcription. A blastn search in RNA-seq reads from libraries of cercariae (panel A) and mixed sex, adult worms (panel B) reported by Protasio and coworkers [32] was performed and a tally of the RNA-seq reads aligning to the four transcripts encoding septins was compared. Values are expressed as number of aligned reads per million of reads per kilobase of transcript. Data for cercariae represent the average from three independent libraries whereas adult worm data were from a single experiment. This analysis revealed a pattern of expression among the four septin genes very similar to that ascertained by the relative qPCR. (TIF) [file pntd.0002602.s004.tif]

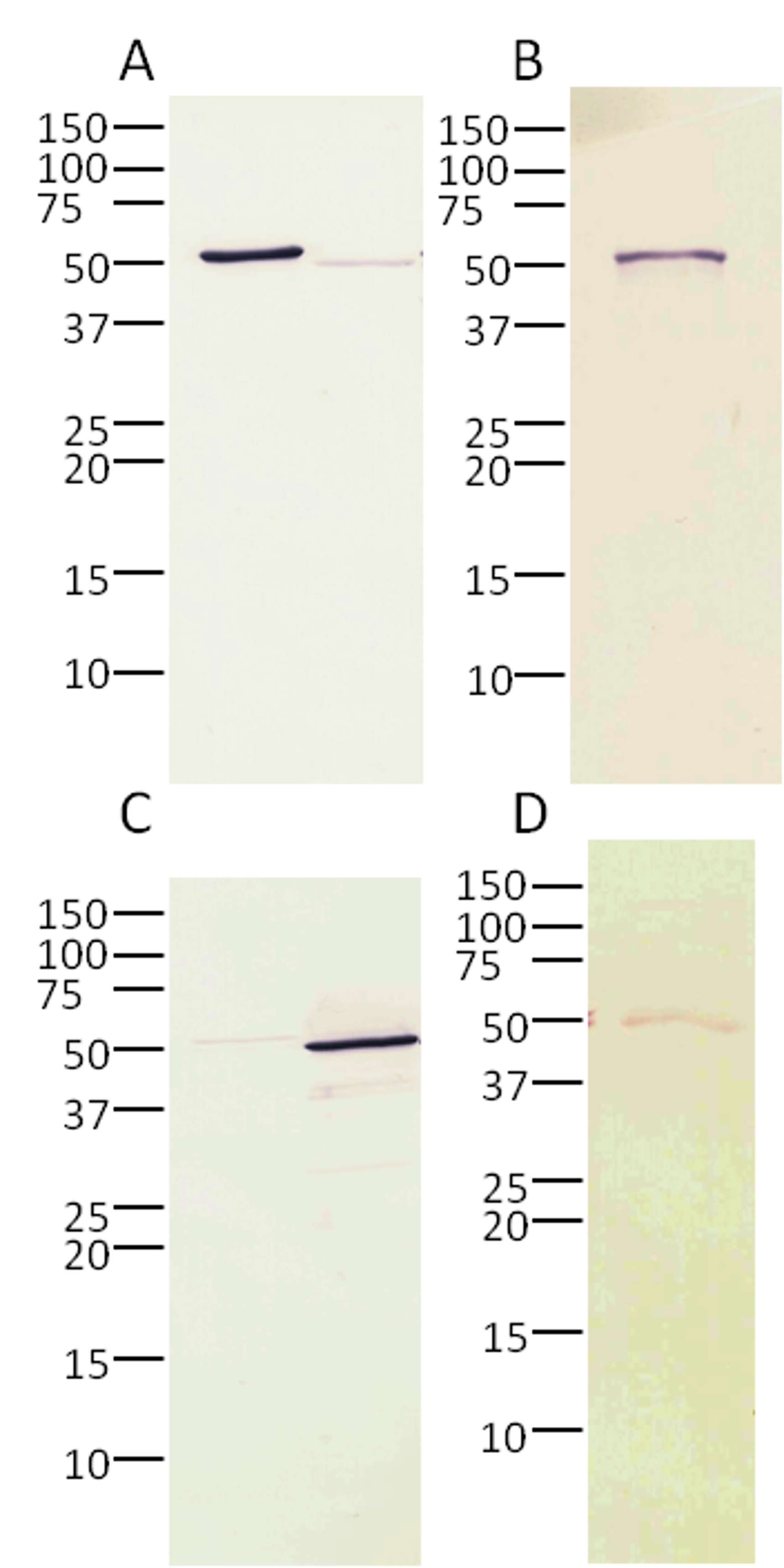

Supplement: Figure S5 — Western blot analysis of S. mansoni septin antibodies. Panel A: Anti-SmSEPT5 recognizes recombinant SmSEPT5 (0.5 µM) at 53.8 kDa (left lane) and weakly recognizes SmSEPT10 (0.5 µM) at 50.5 kDa (left lane). B: Anti-SmSEPT5 recognizes a single band in a lysate of mixed sex adults, at the expected molecular mass. C: Anti-SmSEPT10 recognizes recombinant SmSEPT10 (0.5 µM) (right lane) and weakly recognizes SmSEPT5 (left lane). D: Anti-SmSEPT10 recognizes a single band in a lysate of mixed sex adults, at the expected molecular mass. Molecular size standards in kilodaltons (kDa) are shown at the left of the blots. (TIF) [file pntd.0002602.s005.tif]

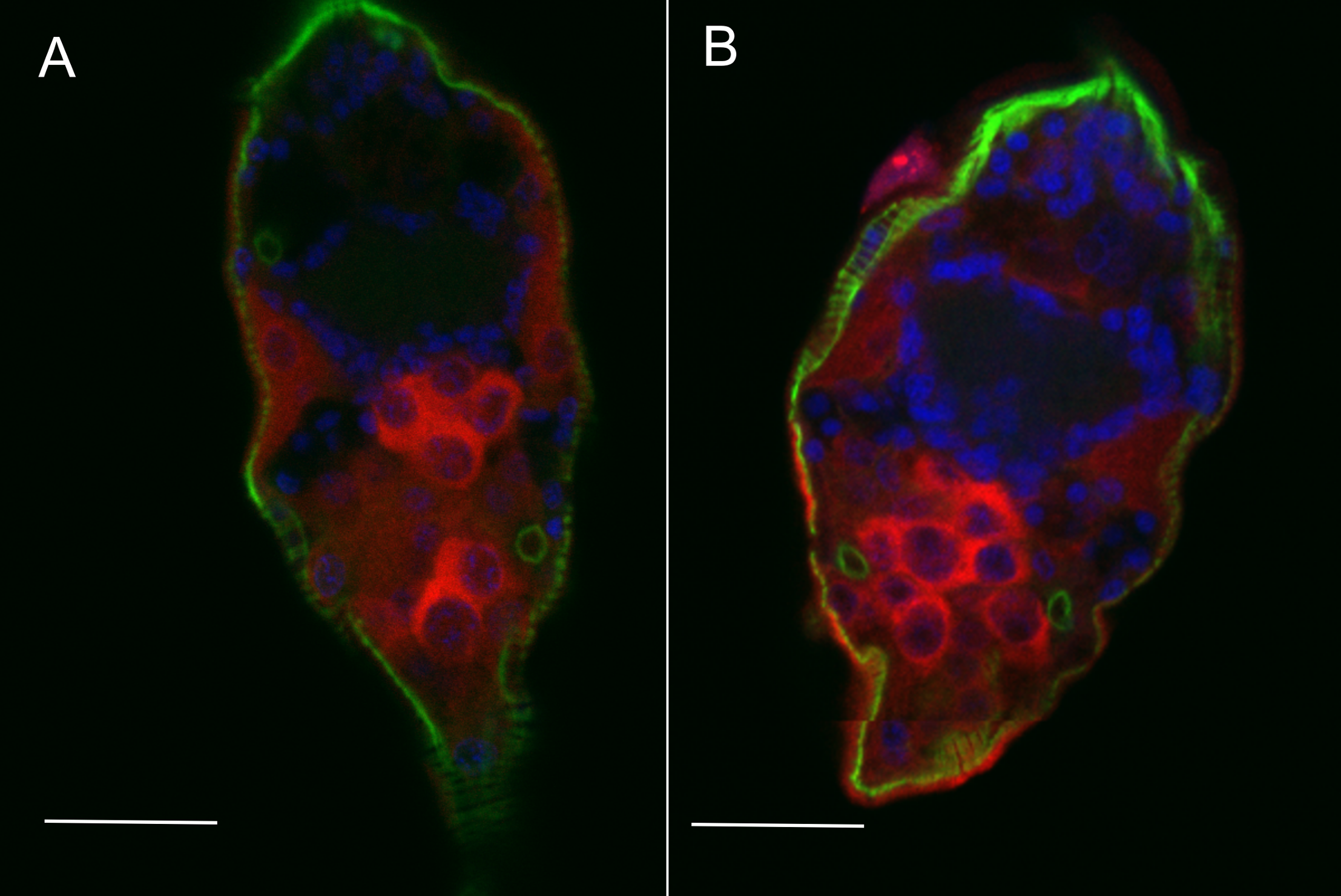

Supplement: Figure S6 — Staining of a miracidium with anti- Sm SEPT5 and anti- Sm SEPT10 immunoglobins. Confocal optical sections of a miracidium labeled with SmSEPT5 (panel A) and anti-SmSEPT10 (B) showing a similar pattern of localization irrespective of which anti-septin probe was deployed. Nuclei stained with DAPI (blue) and actin filaments stained with phalloidin conjugated with Alexa Fluor 568 (green). Probing with both antibodies revealed the prevalence of septin in germ cells of miracidia. Scale bar, 20 µm. (TIFF) [file pntd.0002602.s006.tiff]

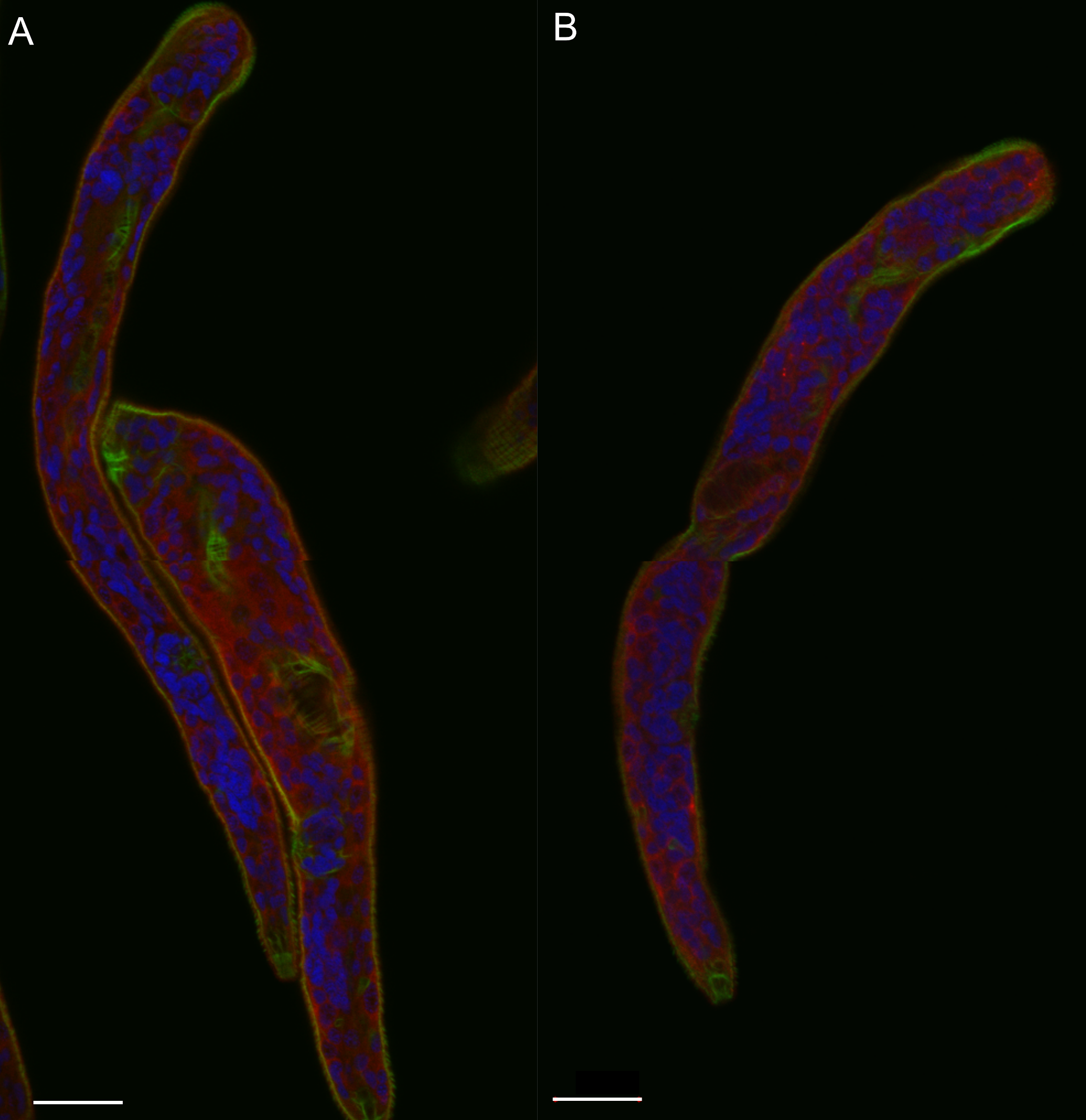

Supplement: Figure S7 — Staining of schistosomula with anti- Sm SEPT5 and - Sm SEPT10 immunoglobulins. Confocal optical sections of schistosomula cultured for 14 days labeled with SmSEPT5 (panel A) or anti-SmSEPT10 (B) immunoglobulins revealed ubiquitous septin localization in this stage for both probes. Nuclei stained with DAPI (blue) and actin filaments stained with phalloidin conjugated with Alexa Fluor 568 (green). Scale bar, 20 µm. (TIFF) [file pntd.0002602.s007.tiff]

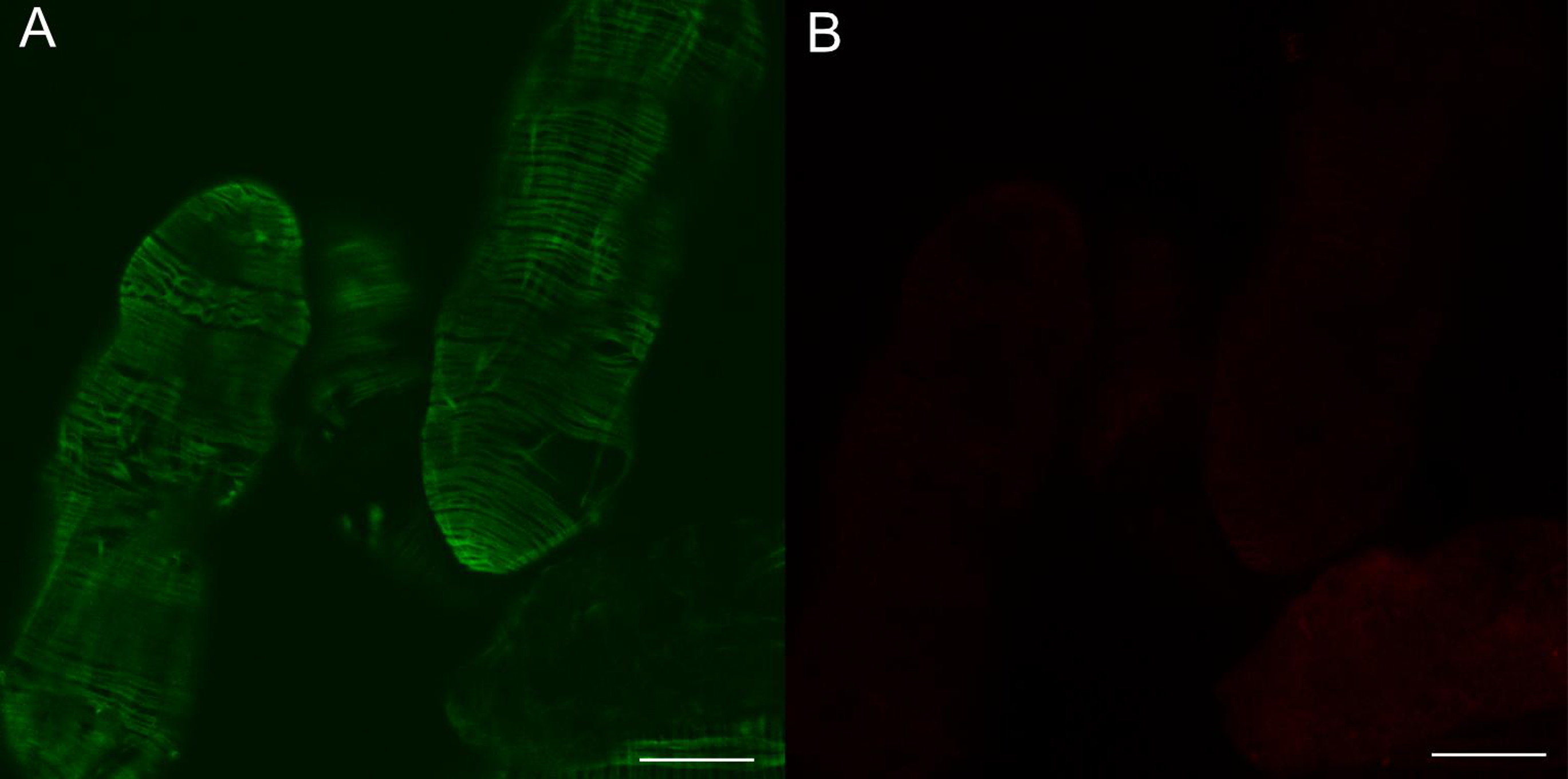

Supplement: Figure S8 — Control samples incubated with secondary antibody. Panel A: F-actin structure stained with phalloidin. B: Miracidia stained only with the secondary antibody conjugated to Alexa Fluor 633. Other developmental stages presented similar background level signals (not shown). (TIF) [file pntd.0002602.s008.tif]

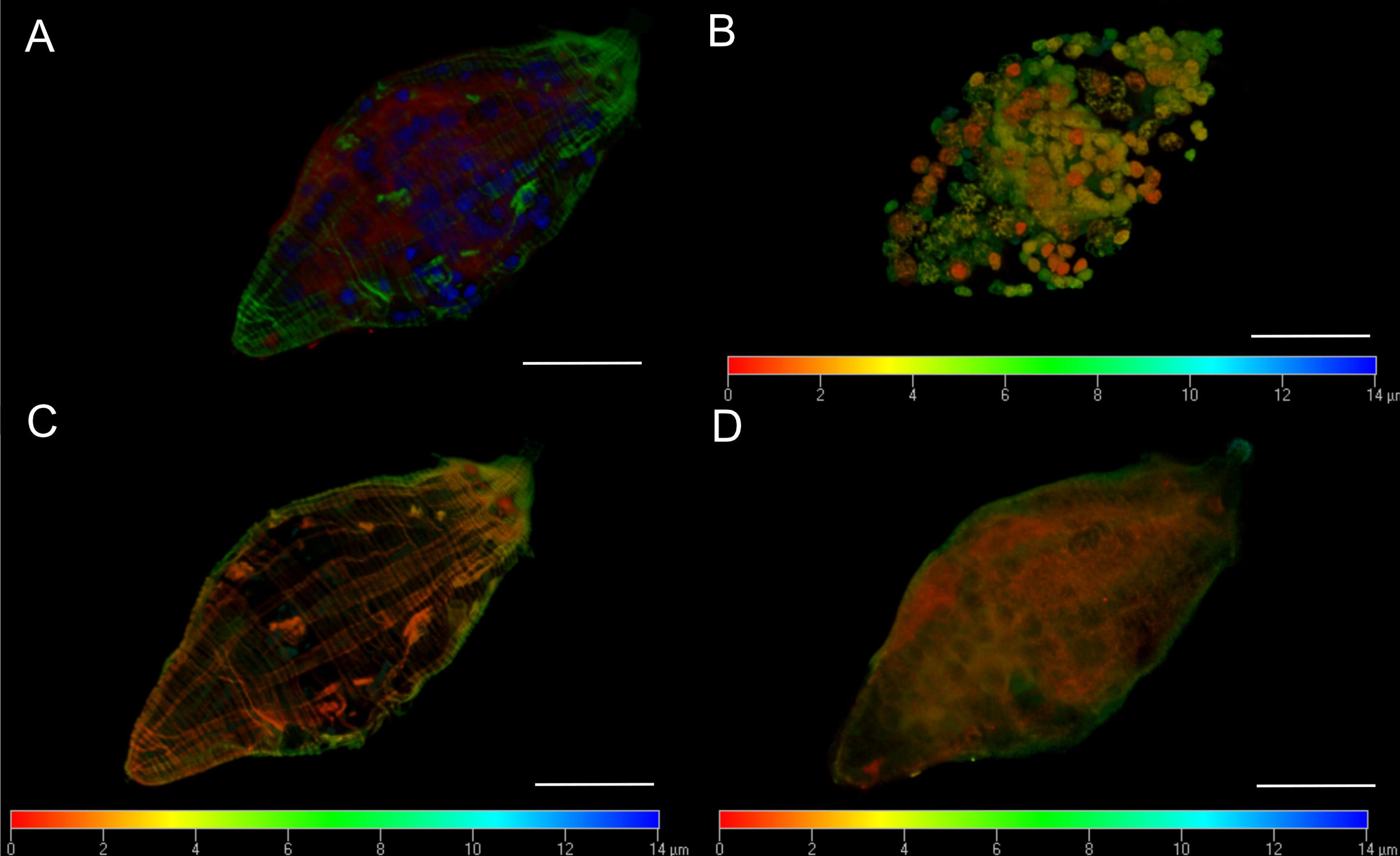

Supplement: Figure S9 — Septins are expressed in the miracidium. Serial optical sections (×28) at 0.5 µm intervals, projected at the z-axis using the Zen software. Panel A: Projections of DAPI (blue), phalloidin (green) and anti-SmSEPT5 (red) signals. B: Individual projection of nuclei stained with DAPI. C: Projection of phalloidin staining several layers of F-actin throughout the miracidium. D: Individual projection of septin labeled with anti-SmSEPT10. The color scale bar represents the depth, and white scale bars represent the length, 20 µm. (TIF) [file pntd.0002602.s009.tif]

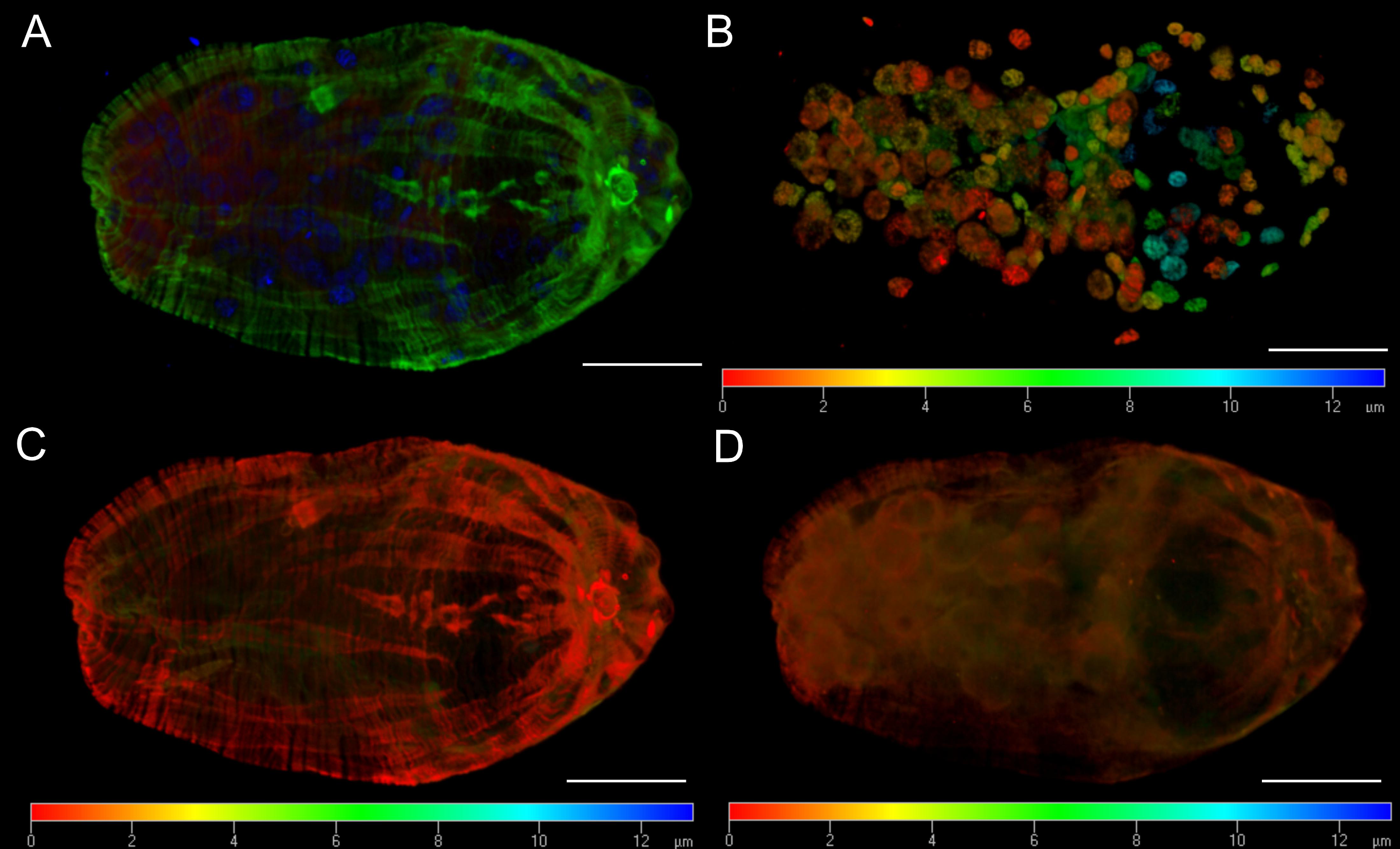

Supplement: Figure S10 — Septins are expressed in the sporocyst. Projection of 28 optical sections of sporocysts cultured for two days. Panel A represents projections of nuclei (blue), actin (green) and septin (red). B: Individual projection of nuclei stained with DAPI. C: Projection of phalloidin staining several layers of F-actin throughout the sporocyst. D: Individual projection of septin labeled with anti-SmSEPT10. The color scale bar represents the depth. White scale bars, the length, 20 µm. (TIF) [file pntd.0002602.s010.tif]
